# Breastfeeding and Infant Temperament at Age Three Months

**DOI:** 10.1371/journal.pone.0029326

**Published:** 2012-01-10

**Authors:** Blandine de Lauzon-Guillain, Katrien Wijndaele, Matthew Clark, Carlo L. Acerini, Ieuan A. Hughes, David B. Dunger, Jonathan C. Wells, Ken K. Ong

**Affiliations:** 1 Medical Research Council Epidemiology Unit, Cambridge, United Kingdom; 2 INSERM, Université Paris-Sud, Villejuif, France; 3 Clinical Medical School, University of Cambridge, Cambridge, United Kingdom; 4 Department of Paediatrics, University of Cambridge, Cambridge, United Kingdom; 5 Childhood Nutrition Research Centre, Institute of Child Health, University College London, London, United Kingdom; Indiana University, United States of America

## Abstract

**Background & Methods:**

To examine the relationship between breastfeeding and maternally-rated infant temperament at age 3 months, 316 infants in the prospective Cambridge Baby Growth Study, UK had infant temperament assessed at age 3 months by mothers using the Revised Infant Behavior Questionnaire, which produces scores for three main dimensions of temperament derived from 14 subscales. Infant temperament scores were related to mode of infant milk feeding at age 3 months (breast only; formula milk only; or mixed) with adjustment for infant's age at assessment and an index of deprivation.

**Results:**

Infant temperament dimension scores differed across the three infant feeding groups, but appeared to be comparable between exclusive breast-fed and mixed-fed infants. Compared to formula milk-fed infants, exclusive breast-fed and mixed-fed infants were rated as having lower impulsivity and positive responses to stimulation (adjusted mean [95% CI] “*Surgency/Extraversion*” in formula-fed vs. mixed-fed vs. breast-fed groups: 4.3 [4.2–4.5] vs. 4.0 [3.8–4.1] vs. 4.0 [3.9–4.1]; p-heterogeneity = 0.0006), lower ability to regulate their own emotions (“*Orienting/Regulation*”: 5.1 [5.0–5.2], vs. 4.9 [4.8–5.1] vs. 4.9 [4.8–5.0]; p = 0.01), and higher emotional instability (“*Negative affectivity*”: 2.8 [2.6–2.9] vs. 3.0 [2.8–3.1] vs. 3.0 [2.9–3.1]; p = 0.03).

**Conclusions:**

Breast and mixed-fed infants were rated by their mothers as having more challenging temperaments in all three dimensions; particular subscales included greater distress, less smiling, laughing, and vocalisation, and lower soothability. Increased awareness of the behavioural dynamics of breastfeeding, a better expectation of normal infant temperament and support to cope with difficult infant temperament could potentially help to promote successful breastfeeding.

## Introduction

There is an overwhelming literature in favour of breastfeeding. The range of benefits of breastfeeding includes protection against infection, allergy, atopy and childhood obesity [1]. Breastfeeding is also associated with better cognitive development [2], [3]. Despite its many benefits and universal promotion, rates of successful breastfeeding remain low in many western populations. According to the Infant Feeding Survey 2005, 76% of UK mothers initiated breastfeeding, but only 48% were still breastfeeding at age six weeks, and only 35% at age four months [4]. Although the promotion of breastfeeding is widespread, little attention has been directed to understanding the modifiable factors that may discourage the practice. The incidence and duration of breastfeeding may depend on a complex mosaic of physical, behavioural, social and economic factors [5],[6] and may also include parental impressions of the infants needs. A number of studies have investigated maternal factors associated with breastfeeding [5], [7], [8], including attempts to understand why mothers cease breastfeeding [9]. While mothers most commonly cite the perception that their infant was not satisfied by breast milk alone as is a key reason for stopping breastfeeding [9], variability in infant behavioural characteristics has received little investigation.

Both irritability and physical sucking are key means whereby mammalian infants signal their hunger to mothers in the first months after birth [10]. Models of signalling treat the mother and offspring as two parties in a dynamic relationship, whereby the level of infant signalling is a function both of its own hunger, and the rate supply of food from the mother [10]. Infants of malnourished mothers have been shown to suckle more often to compensate for the slow transfer of breast-milk [11], while healthy breast-fed infants at 12 weeks were observed to feed more slowly than formula-fed infants despite taking in similar milk volumes, and they also fed more frequently [12]. We hypothesized that such differences in physical signalling of demand may be replicated in the display of greater behavioural signals of hunger in breastfed compared to formula-milk fed infants.

Breastfed neonates have been reported to be more irritable than formula-fed neonates [13], which may indicate a higher level of signalling is required to obtain a given amount of milk. This difference in irritability suggests that the initiation of breastfeeding could be stressful to some mothers and infants. It is possible that continuing difficult temperament in some breastfed infants may contribute to the steady decline in breastfeeding prevalence with increasing infant age. However, previous small studies failed to show any relationship between breastfeeding and infant temperament assessed at age three months by infant behaviour questionnaires or direct observation of mother-infant interaction [12], [14], [15], [16]. Furthermore, in an observational comparison between the control groups of a randomised controlled trial, temperament did not differ between older breastfed or formula-fed infants at age six to twelve months old [17]. As these studies were small, they may have been insufficiently powered to detect differences in infant behaviour relevant to variability in maternal perception of breast-feeding experience.

By administering the Revised Infant Behaviour Questionnaire in a large prospective cohort study, we examined the relationship between breastfeeding and infant temperament at age three months old.

## Materials and Methods

### Study design

The current study is part of a large ongoing birth cohort study examining the prenatal and postnatal determinants of infancy weight gain and adiposity. Inclusion criteria were mothers attending a single antenatal centre in Cambridge UK. Exclusion criteria were mothers aged <16 years, or unable to give informed consent. Mothers were approached and recruited at their first antenatal clinic appointment during early pregnancy by trained paediatric research nurses. The study was approved by the local Cambridge research ethics committee and all mothers gave written informed consent.

At the time of the current analysis, the cohort included 1,526 infants born between August 2001 and June 2009. The current dataset was based on a sub-cohort of 316 infants, born between January 2006 to February 2009, with information on infant temperament and milk-feeding at age 3 months. This sub-sample was representative of the whole cohort with regard to birth weight, mother's BMI, and infant feeding mode at age 3 months (all p>0.2).

### Infant temperament

Infant temperament at the age of three months was assessed by the mother using the Revised Infant Behavior Questionnaire [18]. This 191-item questionnaire provides an assessment of 3 major dimensions of infant temperament: *surgency/extraversion* (higher scores are seen in infants with high activity levels, impulsivity and positive affect in response to highly stimulating situations); *negative affectivity* (higher scores are seen in emotionally less stable infants) and *orienting/regulation* (higher scores are seen in infants with good ability to regulate their own emotions). These dimensions were directly calculated from 14 subscales which each range in score from 1 to 7 (see Appendix S1) [18]. In our cohort, internal consistency reliability coefficients (Cronbach's α) ranged from 0.75 to 0.91 for subscales, and was 0.79 for *surgency/extraversion*, 0.73 for *negative affectivity* and 0.59 for *orienting/regulation* scores.

### Other data collection

Mothers reported their height and pre-pregnancy weight by a self-administered prenatal questionnaire. Maternal pre-pregnancy BMI was calculated as weight/height^2^ (kg/m^2^). We used the “Missing Value Analysis” option in SPSS version 16 to single-impute missing maternal BMI values (n = 35). This imputation was informed by data on maternal age, maternal smoking, gestational diabetes, gestation duration and data on the infant (twin order, sex, and body size at birth). Maternal qualification was also self-recorded in the prenatal questionnaire. The Index of Multiple Deprivation 2007 (IMD) was derived from individual residential postcodes. This index combines a number of indicators that cover a range of economic, social and housing issues into a single deprivation score for each of 32,482 small areas in England [19]. The sample mean IMD value (9.59) was imputed for those cases where it was missing (n = 92) as these mothers did not differ from others with regard to age, BMI, offspring birthweight, gestational age or breastfeeding. Mode of infant milk feeding (breast, formula-milk or mixed-feeding) at age three months was reported by the parents in response to a research nurse administered questionnaire at the three months study visit.

### Statistics

Associations between infant temperament and infant's age and sex were assessed respectively by Pearson's correlations and Student's t-tests. The cross-sectional associations between infant temperament and maternal characteristics were tested by linear regressions, adjusting for infant's age, with infant temperament as the dependant variable. The cross-sectional associations between infant feeding mode and infant temperament were tested by ANCOVA with infant temperament as the dependant variable and adjusting for infant age and Index of Multiple Deprivation (other potential confounders were unrelated to infant temperament; see results below). Statistical package SAS version 9.1 (SAS Institute Inc., Cary, NC, USA) was used for analysis.

## Results

In total, 171 boys and 145 girls with data on temperament at age 3 months were included in this analysis. The mean age of the children when the Revised Infant Behavior Questionnaire was completed was 3.2 months (SD 0.3). Mean (SD) maternal age was 33.2 (4.5) years, mean maternal BMI was 23.8 (2.9) kg/m^2^ and mean gestational age at delivery was 40.0 (1.4) weeks. 137 infants were exclusively breastfed, 88 were exclusively formula-fed and 91 were fed with a mixture of formula and breastfeeding at age 3 months. Characteristics of the study population, according to the mode of feeding are shown in **Table 1**.

**Table 1:** Characteristics of the study population, according to the mode of infant feeding at age 3 months.

	Formula-fed	Mixed-fed	Breastfed
N	88	91	137
Boys (n, %)	48 (55%)	49 (54%)	74 (54%)
Age at 3mo exam (mo)	3.2 (0.3)	3.2 (0.3)	3.2 (0.4)
Firstborn (n, %)	43 (49%)	45 (49%)	67 (49%)
Birth weight (SDS)	0.0 (1.1)	−0.1 (0.9)	0.0 (0.9)
Gestational age (wk)	39.9 (1.4)	39.8 (1.6)	40.2 (1.2)
Maternal BMI (kg/m^2^)	25.1 (4.4)	23.7 (3.4)	23.1 (3.6)
Maternal age (years)	32.5 (4.4)	34.0 (3.9)	33.2 (4.8)
Index of Multiple Deprivation 2007	10.1 (4.2)	8.8 (3.3)	9.8 (3.8)
Maternal Qualification (n, %)			
*Missing	49 (56%)	46 (51%)	85 (62%)
O-level, vocational, other	11 (13%)	5 (5%)	4 (3%)
A-level, Certificate, Diploma	12 (14%)	7 (8%)	7 (5%)
Degree level or higher	16 (18%)	33 (36%)	41 (30%)

Data are mean (SD) or n (%).

*35 mothers did not complete the relevant prenatal questionnaire and 145 did not complete this question.

### Infant temperament according to infant characteristics

None of the 3 main dimensions of infant temperament differed between boys and girls at age 3 months (**Table 2**). Older infants had higher scores for the *surgency/extraversion* dimension (regression coefficient (β)±SE: 0.43±0.12 unit/month, p = 0.0005), but no differences in *negative affectivity* (β±SE: −0.03±0.10 unit/month, p = 0.7) or *orienting/regulation* (β±SE: 0.15±0.09 unit/month, p = 0.1). None of the three infant temperament dimensions was related to infant's birth weight (all p>0.3). Gestational age was not related to *surgency/extraversion* dimension (β±SE: 0.00±0.05 unit/week of gestation, p = 0.7) or to *negative affectivity* (β±SE: −0.02±0.03 unit/week of gestation, p = 0.5) but tended to be negatively related to *orienting/regulation* (β±SE: −0.04±0.02 unit/week of gestation, p = 0.07).

**Table 2:** Infant temperament at age three months according to gender.

	Boys	Girls	
	(n = 171)	(n = 145)	P-value
**Extraversion**	**4.1 (0.8)**	**4.0 (0.8)**	**0.4**
*Activity level*	*3.8 (0.8)*	*3.7 (0.7)*	*0.7*
*Smiling and Laughter*	*4.5 (1.0)*	*4.5 (1.0)*	*1.0*
*High Pleasure*	*5.1 (1.0)*	*5.0 (0.9)*	*0.2*
*Perceptual sensitivity*	*3.4 (1.2)*	*3.3 (1.2)*	*0.6*
*Approach*	*3.8 (1.4)*	*3.7 (1.4)*	*0.6*
*Vocal reactivity*	*4.0 (1.0)*	*3.9 (1.0)*	*0.6*
**Negative affectivity**	**2.9 (0.6)**	**2.9 (0.6)**	**0.7**
*Distress to limitation*	*3.3 (0.8)*	*3.2 (0.8)*	*0.8*
*Fear*	*2.0 (0.6)*	*2.2 (0.8)*	*0.01*
*Falling reactivity*	*5.1 (0.9)*	*5.1 (0.9)*	*0.7*
*Sadness*	*3.5 (1.0)*	*3.5 (1.0)*	*0.8*
**Orienting/Regulation**	**5.0 (0.6)**	**5.0 (0.6)**	**0.5**
*Duration of orienting*	*4.0 (1.1)*	*4.1 (1.1)*	*0.3*
*Low Pleasure*	*5.1 (0.9)*	*5.1 (0.8)*	*0.8*
*Soothability*	*4.8 (0.8)*	*4.9 (0.7)*	*1.0*
*Cuddliness*	*5.9 (0.6)*	*5.9 (0.5)*	*1.0*

Data are means (±SD).

Main infant temperament dimensions are shown in bold and subscales in italics.

### Infant temperament according to maternal characteristics

The Index of Multiple Deprivation (IMD) was positively related to *surgency/extraversion* (β±SE: 0.03±0.01 unit/IMD unit, p = 0.01, adjusted for infant's age) but not to *negative affectivity* (β±SE: 0.01±0.01 unit/IMD unit, p = 0.4) or *orienting/regulation* (β±SE: 0.01±0.01 unit/IMD unit, p = 0.09).

Maternal BMI was not associated with *surgency/extraversion* (β±SE: 0.02±0.01 unit/kg.m^−2^, p = 0.1, adjusted for infant's age), *negative affectivity* (β±SE: −0.00±0.01 unit/kg.m^−2^, p = 0.6) or *orienting/regulation* (β±SE: 0.01±0.01 unit/kg.m^−2^, p = 0.2). Maternal age was not associated with *surgency/extraversion* (β±SE: −0.0−±0.01 unit/kg.m^−2^, p = 0.3, adjusted for infant's age), *negative affectivity* (β±SE: −0.01±0.01 unit/kg.m^−2^, p = 0.2) or *orienting/regulation* (β±SE: 0.00±0.01 unit/kg.m^−2^, p = 0.9). Similarly, maternal qualification was not related to infant temperament (all p>0.3).

### Infant temperament and breastfeeding at age 3 months

**Figure 1:**
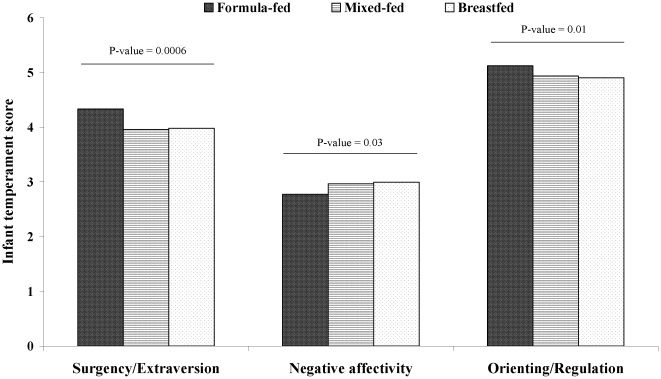
Mean scores for the three dimensions of infant temperament at age 3 months, by mode of infant feeding at age 3 months. P-values are shown for tests of heterogeneity between all groups, adjusted for age.

At age 3 months, scores of the *surgency/extraversion* dimension of infant temperament differed substantially across the three infant feeding groups (ANCOVA: P = 0.0006, adjusted for infant's age, and index of multiple deprivation); breastfed and mixed-fed infants had lower *surgency/extraversion* scores compared to formula-fed infants (**Table 3**). Differences between the three infant feeding groups were also seen for *negative affectivity* (ANCOVA: P = 0.03) and *orienting/regulation* (ANCOVA: P = 0.01) (**Table 3** and **Figure 1**). These differences persisted without any detectable attenuation following adjustment for IMD (**Table 4**).

**Table 3:** Mode of feeding and infant temperament, assessed at age three months (n = 316).

	Formula-fed	Mixed-fed	Breastfed	P-values from heterogeneity tests:	
	N = 88	N = 91	N = 137	Across all 3 feeding groups	Formula-fed versus the others
**Extraversion**	**4.3 (4.2–4.5)**	* **4.0 (3.8–4.1)**	* **4.0 (3.9–4.1)**	***0.0006***	***0.0001***
Activity level	3.8 (3.7–4.0)	3.7 (3.5–3.8)	3.7 (3.6–3.9)	*0.4*	*0.2*
Smiling and Laughter	4.8 (4.6–5.0)	*4.4 (4.2–4.6)	*4.4 (4.2–4.5)	*0.0008*	*0.0002*
High Pleasure	5.4 (5.2–5.6)	*5.0 (4.8–5.2)	*5.0 (4.8–5.1)	*0.006*	*0.002*
Perceptual sensitivity	3.5 (3.3–3.8)	3.2 (3.0–3.5)	3.4 (3.2–3.6)	*0.2*	*0.2*
Approach	4.1 (3.8–4.4)	3.7 (3.4–4.0)	*3.6 (3.4–3.9)	*0.06*	*0.009*
Vocal reactivity	4.4 (4.2–4.6)	*3.8 (3.6–4.0)	*3.8 (3.7–4.0)	*<0.0001*	*<0.0001*
**Negative affectivity**	**2.8 (2.6–2.9)**	**3.0 (2.8–3.1)**	* **3.0 (2.9–3.1)**	***0.03***	***0.007***
Distress to limitation	3.0 (2.9–3.2)	3.3 (3.2–3.5)	*3.3 (3.2–3.5)	*0.02*	*0.005*
Fear	2.1 (1.9–2.2)	2.1 (1.9–2.2)	2.1 (1.9–2.2)	*1.0*	*0.9*
Falling reactivity	5.3 (5.2–5.5)	*5.0 (4.8–5.2)	*5.0 (4.8–5.1)	*0.008*	*0.002*
Sadness	3.3 (3.1–3.5)	3.5 (3.3–3.7)	3.6 (3.4–3.7)	*0.2*	*0.08*
**Orienting/Regulation**	**5.1 (5.0–5.2)**	* **4.9 (4.8–5.1)**	* **4.9 (4.8–5.0)**	***0.01***	***0.004***
Duration of orienting	4.2 (4.0–4.4)	4.0 (3.8–4.2)	3.9 (3.8–4.1)	*0.2*	*0.06*
Low Pleasure	5.3 (5.1–5.5)	5.1 (5.0–5.3)	*5.0 (4.8–5.1)	*0.05*	*0.04*
Soothability	5.1 (5.0–5.3)	*4.8 (4.6–4.9)	*4.7 (4.6–4.8)	*0.0001*	*<0.0001*
Cuddliness	5.9 (5.8–6.0)	5.8 (5.7–6.0)	5.9 (5.8–6.0)	*0.6*	*1.0*

Data are estimated marginal means (95% CI), adjusted for infant's age and index of multiple deprivation.

Main infant temperament dimensions are shown in bold.

*P<0.05 versus the Formula-fed group.

**Table 4:** Univariate and multivariate associations between mode of feeding and Index of Multiple Deprivation (IMD) on infant temperament.

	Surgency/Extraversion		Negative affectivity		Orienting/Regulation	
	B (SE)	*P-value*	B (SE)	*P-value*	B (SE)	*P-value*
Univariate models:						
Mode of feeding at 3mo		*0.0003*		*0.03*		*0.01*
Formula-fed	0.36 (0.1)		−0.22 (0.08)		0.36 (0.1)	
Mixed-fed	−0.04 (0.1)		−0.04 (0.08)		−0.04 (0.1)	
Breast-fed	0 (Ref)		0 (Ref)		0 (Ref)	
IMD	0.03 (0.01)	*0.01*	0.01 (0.01)	*0.4*	−0.04 (0.1)	*0.09*
Multivariate model:						
Mode of feeding at 3mo		*0.0006*		*0.03*		*0.01*
Formula-fed	0.35 (0.1)		−0.22 (0.08)		0.36 (0.1)	
Mixed-fed	−0.02 (0.1)		−0.03 (0.08)		−0.04 (0.1)	
Breast-fed	0 (Ref)		0 (Ref)		0 (Ref)	
IMD	0.02 (0.01)	*0.03*	0.01 (0.01)	*0.4*	−0.04 (0.1)	*0.1*

B: regression coefficient.

Mean scores for the main dimensions and subscales of infant temperament are presented for each of the three infant feeding groups in **Table 3**. Breastfed and mixed-fed infants had lower scores in *surgency/extraversion* and *orienting/regulation* compared to formula-fed infants. Breastfed infants also had higher scores in *negative affectivity* compared to formula-fed infants. Breastfed and mixed-fed infants had very similar mean scores for the main dimensions and subscales of infant temperament and, compared to formula-fed infants, both these groups had lower scores for *smiling and laughter*, *high pleasure*, *vocal reactivity* and *soothability*, but higher scores for *falling reactivity* (slower rate of recovery from distress or arousal). In addition, compared to formula-fed infants, breast-fed infants had lower scores for *approach* (positive anticipation of pleasurable activities) and *low pleasure* (enjoyment related to low stimulus intensity) and higher scores for *distress to limitation* (greater fussing, crying or showing distress).

## Discussion

In our UK birth cohort study, infants who were breastfed or mixed-fed at three months of age were rated by their mothers as having overall more challenging temperaments, with lower scores for *surgency/extraversion* and *orienting/regulation*, and higher scores for *negative affectivity* compared to formula-fed infants. Consistent differences between these groups were seen across many of the subscales that contribute to the main infant temperament dimensions. In particular, compared to formula-fed infants, breastfed infants were reported to show greater distress, less smiling, laughing and vocalisation, to be slower to calm down following distress or excitement, and more difficult to soothe by caregivers.

Humans often perceive infant crying as stress, but for infant animals irritability is a normal component of signalling to parents. The expression of offspring demand is part of a dynamic signalling system between parents and offspring, and has received much attention from zoologists studying a variety of bird and mammal species [10]. Zoologists assume that offspring transmit signals of nutritional need, and that parents respond with an appropriate transfer of food. Considerable effort has been invested in understanding how this dynamic relationship functions, for example how each party can avoid manipulation by the other. According to theoretical principles, ‘honest’ signals should be metabolically expensive, because under these circumstances, it is more costly to transmit false information than true information [20]. Consistent with this prediction, an experimental study showed that chicks forced to beg more to obtain their food, by artificially inflating their signals of demand, suffered a penalty in growth rate [21].

Similar components of signalling systems have been demonstrated in human infants [10]. In the first months of infancy, the metabolic costs of crying are 20 times those of sleep [22], and at 12 weeks irritability is a significant determinant of total daily energy expenditure [23]. Consistent with the study of birds, the marginal cost of crying is also high in early infancy, as 40% of energy is directed to growth at 6 weeks, and 30% at 12 weeks [24]. However, these marginal costs subsequently decline, as by 6 months only 10% of energy is required for growth [24] and there is therefore less energetic constraint on crying. Crying and irritability are therefore predicted to act more as an honest signal of nutritional need during earlier than later infancy, and this could explain the lack of difference in temperament between older breastfed and formula-fed infants at age six to twelve months old [17].

Previous studies comparing infant temperament between breastfed and formula-fed infants have shown mixed results. Neonates appear to experience the initiation of breastfeeding as more stressful than the initiation of formula-feeding, as suggested by observations of greater irritability [13] and more frequent crying/fussing behaviour [25] in breastfed newborns compared to formula-fed newborns. With this in mind, it is not surprising that more challenging temperaments were seen in breastfed infants at age three months in our study. However, previous studies among three-month-old infants found no associations between breastfeeding and infant temperament [12], [14], [16]. This discrepancy could be due to the larger sample size of our study; those earlier studies each involved less than 60 infants. Secondly, infant temperament was assessed in our study by the IBQ-R whereas it was assessed by the earlier IBQ in two previous studies [12], [16]. Compared to the IBQ additional scales were created for the IBQ-R, including *approach, high pleasure index, perceptual sensitivity* and *vocal reactivity*. The addition of these new subscales could have strengthened the association between mode of feeding and the temperament dimensions. In the other previous study [14], infant temperament was assessed by observation, and included only “fussy/crying” and “time to calm” dimensions that are not directly assessed by the IBQ-R.

In longitudinal studies, longer breastfeeding duration seemed to be associated with easier perceived infant temperaments. In 50 infants followed from birth to 12 weeks, breastfeeding duration was associated with reported ‘easy’ temperament [26], evaluated by the Infant Characteristics Questionnaire developed by Bates et al. [27]. Similarly, using the same questionnaire, Niegel et al. reported [28] in 30,466 infants, followed from birth to 18 months, that fussy/difficult temperament was related to lower rate of exclusive breastfeeding only at age 6 months. One explanation for the discrepancy between cross-sectional and longitudinal studies could be survivor bias, i.e. breastfeeding may be causally related to more difficult infant temperament, but among infants who are initially breastfed, those with easier temperaments might be more likely to remain breastfed for longer. Further longitudinal studies of breastfed infants are required to assess whether infant temperament assessed in early infancy is a predictor of breastfeeding duration.

Although this is not a large study, our sample size was larger than most previous studies of infant temperament and breastfeeding [12], [13], [14], [16], [17], [26]. We acknowledge that the sample was not designed to be representative of UK infants, although our population had similar birth weights to the British 1990 growth reference. The average IMD value of in our sample 9.59 (range 3.59 to 25.10) is representative of the local Cambridgeshire County (average IMD 11.49) from which it is drawn, but is less deprived compared to many of the other 149 English counties where average IMD varies from 5.36 to 46.97. The use of the IBQ-R allowed us to examine a wide range of behaviours, with nine additional scales compared to the original IBQ [18]. Infant temperament was subjectively rated in our study by mothers, and unfortunately we did not collect data on mother-child interactions such as mother's working, time spent with infants, feeding schedules and other daily structures to see if these behavioural factors might influence the ratings or modify the effects of infant feeding. However Gartstein and colleagues found a moderate agreement between ratings given by primary and secondary caregivers [18]. The IBQ-R provides a differentiated continuous measure of infant temperament, emphasizing both reactive and regulatory capacities, but does not identify thresholds for difficult temperament [18]. However, normal variations in infant temperament are closely related to parenting stress and anxiety in mothers [29], [30]. Furthermore, various subscales of early infant temperament up to age 12 months old, but not temperament at ages 18 and 36 months [31], have also been shown to be predictive of infant growth rates [32], weight status and body fatness up to age 6 years [31], [33], [34]. We also acknowledge that mode of infant milk feeding at age 3 months was reported by the parents in response to a nurse-administered questionnaire and the validity of these responses was not tested.

Finally, findings from such observational studies do not provide evidence for causality. We were able to consider and where necessary make adjustments for potential confounding by infant sex and age, and by maternal BMI, education and deprivation. We observed that older infants had higher scores for *surgency/extraversion*, which is in keeping with previous reports of a continuity of temperament change from infancy to mid-childhood [18], [35], and we therefore adjusted our models for infant age. Previous studies found only limited sex differences in infant temperament [18], [36], and we were unable to confirm such differences in our cohort. Moreover, only one study had examined associations between children's temperament and parental BMI [37] and only few previous studies had examined associations between infant temperament and socio-economic status, indicating no or limited association with maternal education and family income [38], [39]. We observed that infant *surgency/extraversion* was positively associated with IMD, which is a geographical index of deprivation, however these associations were independent of infant feeding groups (**Table 4**). Compared to the large differences seen in maternal education, mean IMD values differed only modestly between infant feeding groups (**Table 1**), possibly because it is a less precise determinants of feeding choices. Difficult infant temperament has also been associated with greater parental stress, anxiety and depression [29], [30], [40]. We did not have information on these psychological factors, which have previously been negatively associated with breastfeeding [5], [6], and may therefore potentially attenuate the sizes of our observed associations.

In conclusion, our findings indicate that breastfeeding may be demanding for mothers and infants. Breastfed and mixed-fed infants were rated as having overall more challenging temperaments, with lower scores for positive emotionality, lower ability to regulate their own emotions, and lower emotional stability than formula-fed infants. These findings should not be taken to discourage mothers to breastfeed, but rather may suggest new potential avenues to improve breast-feeding duration. In particular, mothers who breastfeed may perceive that other people's formula fed babies are more content, and evidence suggests that some mothers believe that the main cause of infant distress is hunger [41]. The most consistent reason given for women to stop breastfeeding is that “Breast milk alone didn't satisfy my baby” [9], which reflects mother's perception of signalling by the infant. Mothers could receive more information about the behavioural dynamics of breastfeeding so as to have a better expectation and understanding of normal infant temperament and, where necessary, support to cope with difficult aspects of infant temperament.

## Supporting Information

Appendix S1 **The three major dimensions of Infant Temperament and their subscales as assessed by the Infant Behaviour Questionnaire – Revised** [**11**] **.**
(DOC)

## References

[1] Yngve A, Kylberg E, Sjostrom M (2001). Breast-feeding in Europe–rationale and prevalence, challenges and possibilities for promotion.. Public Health Nutr.

[2] Fergusson DM, Beautrais AL, Silva PA (1982). Breast-feeding and cognitive development in the first seven years of life.. Soc Sci Med.

[3] Horwood LJ, Fergusson DM (1998). Breastfeeding and later cognitive and academic outcomes.. Pediatrics.

[4] Bolling K, Grant C, Hamlyn B, Thornton A, Service GS (2007). Infant Feeding Survey 2005.. Information Centre.

[5] Thulier D, Mercer J (2009). Variables associated with breastfeeding duration.. J Obstet Gynecol Neonatal Nurs.

[6] Whalen B, Cramton R (2010). Overcoming barriers to breastfeeding continuation and exclusivity.. Curr Opin Pediatr.

[7] Sullivan ML, Leathers SJ, Kelley MA (2004). Family characteristics associated with duration of breastfeeding during early infancy among primiparas.. J Hum Lact.

[8] Birch LL (2006). Child Feeding Practices and the Etiology of Obesity.. Obesity.

[9] Li R, Fein SB, Chen J, Grummer-Strawn LM (2008). Why mothers stop breastfeeding: mothers' self-reported reasons for stopping during the first year.. Pediatrics.

[10] Wells JC (2003). Parent-offspring conflict theory, signaling of need, and weight gain in early life.. Q Rev Biol.

[11] Delgado HL, Martorell R, Klein RE (1982). Nutrition, lactation, and birth interval components in rural Guatemala.. Am J Clin Nutr.

[12] Wells JC, Davies PS (1995). Diet and behavioural activity in 12-week-old infants.. Ann Hum Biol.

[13] DiPietro JA, Larson SK, Porges SW (1987). Behavioral and heart rate pattern differences between breast-fed and bottle-fed neonates.. Developmental Psychology.

[14] Crockenberg SB, Smith P (1982). Antecedents of mother-infant interaction and infant irritability in the first three months of life.. Infant Behavior and Development.

[15] Worobey J (1993). Effects of feeding method on infant temperament.. Adv Child Dev Behav.

[16] Worobey J (1998). Feeding method and motor activity in 3-month-old human infants.. Percept Mot Skills.

[17] Auestad N, Halter R, Hall RT, Blatter M, Bogle ML (2001). Growth and development in term infants fed long-chain polyunsaturated fatty acids: a double-masked, randomized, parallel, prospective, multivariate study.. Pediatrics.

[18] Gartstein MA, Rothbart MK (2003). Studying infant temperament via the Revised Infant Behavior Qusestionnaire.. Infant Behavior & Development.

[19] Communities and Local Government (2007). The English Indices of Deprivation.

[20] Godfray HC (1995). Evolutionary theory of parent-offspring conflict.. Nature.

[21] Kilner RM (2001). A growth cost of begging in captive canary chicks.. Proc Natl Acad Sci U S A.

[22] Thureen PJ, Phillips RE, Baron KA, DeMarie MP, Hay WW (1998). Direct measurement of the energy expenditure of physical activity in preterm infants.. J Appl Physiol.

[23] Wells JCK, Davies PSW (1996). Relationship between behavior and energy expenditure in 12-week-old infants.. American Journal of Human Biology.

[24] Wells JC, Davies PS (1998). Estimation of the energy cost of physical activity in infancy.. Arch Dis Child.

[25] Barr RG, Kramer MS, Pless IB, Boisjoly C, Leduc D (1989). Feeding and temperament as determinants of early infant crying/fussing behavior.. Pediatrics.

[26] Vandiver TA (1997). Relationship of mothers' perceptions and behaviors to the duration of breastfeeding.. Psychol Rep.

[27] Bates JE, Freeland CA, Lounsbury ML (1979). Measurement of infant difficultness.. Child Dev.

[28] Niegel S, Ystrom E, Hagtvet KA, Vollrath ME (2008). Difficult temperament, breastfeeding, and their mutual prospective effects: the Norwegian Mother and Child Cohort Study.. J Dev Behav Pediatr.

[29] Britton JR (2011). Infant temperament and maternal anxiety and depressed mood in the early postpartum period.. Women Health.

[30] Gray PH, Edwards DM, O'Callaghan MJ, Cuskelly M (2011). Parenting stress in mothers of preterm infants during early infancy.. Early Hum Dev.

[31] Slining MM, Adair L, Goldman BD, Borja J, Bentley M (2009). Infant temperament contributes to early infant growth: A prospective cohort of African American infants.. Int J Behav Nutr Phys Act.

[32] Burton P, Wells JC, Kennedy K, Nicholl R, Khakoo A (2011). Association between infant correlates of impulsivity - surgency (extraversion) - and early infant growth.. Appetite.

[33] Wells JC, Stanley M, Laidlaw AS, Day JM, Stafford M (1997). Investigation of the relationship between infant temperament and later body composition.. Int J Obes Relat Metab Disord.

[34] Faith MS, Hittner JB (2010). Infant temperament and eating style predict change in standardized weight status and obesity risk at 6 years of age.. Int J Obes (Lond).

[35] Komsi N, Raikkonen K, Pesonen AK, Heinonen K, Keskivaara P (2006). Continuity of temperament from infancy to middle childhood.. Infant Behav Dev.

[36] Parade SH, Leerkes EM (2008). The reliability and validity of the Infant Behavior Questionnaire-Revised.. Infant Behav Dev.

[37] Agras WS, Hammer LD, McNicholas F, Kraemer HC (2004). Risk factors for childhood overweight: a prospective study from birth to 9.5 years.. J Pediatr.

[38] Austin MP, Hadzi-Pavlovic D, Leader L, Saint K, Parker G (2005). Maternal trait anxiety, depression and life event stress in pregnancy: relationships with infant temperament.. Early Hum Dev.

[39] Jansen PW, Raat H, Mackenbach JP, Jaddoe VW, Hofman A (2009). Socioeconomic inequalities in infant temperament: the generation R study.. Soc Psychiatry Psychiatr Epidemiol.

[40] Hanington L, Ramchandani P, Stein A (2010). Parental depression and child temperament: assessing child to parent effects in a longitudinal population study.. Infant Behav Dev.

[41] Redsell SA, Atkinson P, Nathan D, Siriwardena AN, Swift JA (2010). Parents' beliefs about appropriate infant size, growth and feeding behaviour: implications for the prevention of childhood obesity.. BMC Public Health.

