# Supplementary material for: Breastfeeding and Infant Temperament at Age Three Months
Source: PLoS One. 2012 Jan 10;7(1):e29326. doi: 10.1371/journal.pone.0029326 (PMC3254612; doi:10.1371/journal.pone.0029326)
Supplement: Appendix S1 — The three major dimensions of Infant Temperament and their subscales as assessed by the Infant Behaviour Questionnaire – Revised [11] . (DOC) [file pone.0029326.s001.doc]

**Appendix S1:** The three major dimensions of Infant Temperament and their subscales as assessed by the Infant Behaviour Questionnaire – Revised [11]

| **Surgency/Extraversion** |  |
| --- | --- |
| Approach | Approach Rapid approach, excitement, and positive anticipation of pleasurable activities. (“When given a new toy, how often did the baby get very excited about getting it?”) |
| High Pleasure | Pleasure or enjoyment related to high stimulus intensity, rate, complexity, novelty, and incongruity. (“During a peek-a-boo game, how often did the baby smile?”) |
| Smiling and Laughter | Smiling or laughter during general caretaking and play. (“How often during the last week did the baby smile or laugh when given a toy?”) |
| Physical activity | Gross motor activity, including movement of arms and legs, squirming and locomotor activity. (“When put into the bath water, how often did the baby splash or kick?”) |
| Vocal reactivity | Amount of vocalization exhibited by the baby in daily activities. (“When being dressed undressed during the last week, how often did the baby coo or vocalize?”) |
| Perceptual Sensitivity | Sensitivity Detection of slight, low intensity stimuli from the external environment. (“How often did the baby notice fabrics with scratchy texture (e.g., wool)?”) |
| **Negative Affectivity** |  |
| Sadness | Lowered mood and activity related to personal suffering, physical state, object loss, or inability to perform a desired action; general low mood. (“Did the baby seem sad when the caregiver was gone for an unusually long period of time?”) |
| Distress to limitation | Fussing, crying or showing distress while (a) in a confining place or position; (b) in caretaking activities; (c) unable to perform a desired action. (“When placed on his/her back, how often did the baby fuss or protest?”) |
| Fear | Startle or distress to sudden changes in stimulation, novel physical objects or social stimuli; inhibited approach to novelty. (“How often during the last week did the baby startle to a sudden or loud noise?”) |
| Falling reactivity (loading negatively) | Rate of recovery from peak distress, excitement, or general arousal; ease of falling asleep. (“When frustrated with something, how often did the baby calm down within 5 min?”) |
| **Orienting/Regulation** |  |
| Low Pleasure | Amount of pleasure or enjoyment related to low stimulus intensity, rate, complexity, novelty and incongruity. (“When playing quietly with one of his/her favourite toys, how often did the baby show pleasure?”) |
| Cuddliness | Expression of enjoyment and moulding of the body to being held by a caregiver. (“When rocked or hugged, during the last week, how often did the baby seem to enjoy him/herself?”) |
| Soothability | Reduction of fussing, crying, or distress when soothing techniques are used by the caregiver. (“When patting or gently rubbing some part of the baby’s body, how often did s/he soothe immediately?”) |
| Duration of Orienting | Attention to and/or interaction with a single object for extended periods of time. (“How often during the last week did the baby stare at a mobile, crib bumper or picture for 5 min or longer?”) |
